# Febrile temperatures increase *in vitro* antibody affinity for malarial and dengue antigens

**DOI:** 10.1371/journal.pntd.0007239

**Published:** 2019-04-03

**Authors:** Razvan C. Stan, Katia S. Françoso, Rubens P. S. Alves, Luís Carlos S. Ferreira, Irene S. Soares, Maristela M. de Camargo

**Affiliations:** 1 Department of Immunology, Institute for Biomedical Sciences, University of São Paulo, Brazil; 2 Department of Clinical and Toxicological Analysis, School of Pharmaceutical Sciences, University of São Paulo, Brazil; 3 Department of Microbiology, Institute for Biomedical Sciences, University of São Paulo, Brazil; Fundacao Oswaldo Cruz, BRAZIL

## Abstract

Fever is a regulated increase of the body temperature resulting from both infectious and non-infectious causes. Fever is known to play a role in modulating immune responses to infection, but the potential of febrile temperatures in regulating antigen binding affinity to antibodies has not been explored. Here we investigated this process under *in vitro* conditions using Isothermal titration calorimetry and ELISA. We used selected malarial and dengue antigens against specific monoclonal antibodies, and observed a marked increase in the affinity of these antibody-antigen complexes at 40°C, compared to physiological (37°C) or pathophysiological temperatures (42°C). Induced thermal equilibration of the protein partners at these temperatures *in vitro*, prior to measurements, further increased their binding affinity. These results suggest another positive and adaptive role for fever *in vivo*, and highlight the favourable role of thermal priming in enhancing protein-protein affinity for samples with limited availability.

## Author summary

Infections with dengue viruses and malaria protozoans affect approximately a third of the world´s population and their epidemiologic patterns constitute a burden for the human health and the global economy. Even though infections with these parasites are often asymptomatic, it a spectrum of physiologic responses can be elicited from the human hosts, including fever. We explore here a hitherto unknown beneficial role of febrile temperatures, namely the increase in antibody affinity for relevant antigens from these pathogens at 40°C, compared to the physiologic body temperature. We also highlight the importance of temperature equilibration prior to protein-protein measurements *in vitro*. We anticipate that these results are useful for delineating the role of febrile temperatures in the immune response against infections.

## Introduction

Maintaining a constant temperature in mammals is a tightly regulated process, including when where infections occur and the body temperature increases. Fever, which is an initial, nonspecific, acute-phase response to infections, is a key factor in improving survival and shortening disease duration [1]. Cellular events occurring during physiological fever or hyperpyrexia have been the focus of intensive clinical and *in vitro* studies [2]. Fever-inducing pathogen load is reduced mainly due to enhanced host defence, while pathogen proliferation at febrile temperatures is not significantly affected [3]. Physiological and reversible increase in core body temperature is not normally higher than 40°C [4], with survival chances beginning to decrease when fever exceeds 39.5°C, suggesting the existence of an upper limit for the optimal fever range [5].

Antibodies progressively mature their affinity and specificity for various target antigens by changing the amino acid residue composition of their complementarity-determining regions [6]. As with other proteins, high affinity for substrates is achieved by fast association rates coupled to slow off-rates in a process directly dependent, among other factors, on temperature. The thermal optimum of antibody-antigen complex depends thus on the chemical nature of the epitope and paratope, and on the type of bonds formed at different temperatures [7].

Recent results using cell-based assays described an increase of the association rates between two monoclonal antibodies to receptors from cancer cells, within a temperature range of 15°C to 37°C [8]. While elevated temperatures greatly alter membrane fluidity, cell signalling and gene expression patterns, the role of febrile temperatures in directly affecting antibody affinity for antigens from pathogens that induce fever has not been explored.

We have focused here on the *in vitro* changes in binding affinities for two antibody-antigen immune complexes of two widespread, fever-inducing infectious diseases [9, 10]. To this end, we made use of antigens from a viral agent, *i*.*e*. non-structural protein 1 (NS1) from dengue virus serotype 2 DENV-2 [11], and from a protozoan pathogen, namely the 19-kDa carboxy-terminal region of merozoite surface protein 1 (MSP1_19_) from *Plasmodium vivax* [12], and their corresponding monoclonal IgG antibodies [13, 14]. We measured a peak in the affinity constant of the protein partners at 40°C, largely due to an entropic contribution to binding, followed by a notable decrease at 42°C, possibly due to protein unfolding. These results may be relevant for unravelling the physiological mechanisms that are activated during fever-inducing infections.

## Materials and methods

### ELISA measurements with dengue DENV-2 antigen and antibody

ELISA with solid-phase bound NS1 protein was carried out as previously described [11]. Briefly, polystyrene Maxisorp microplates (Nunc) were coated overnight at 37°C, 40°C or 42°C with a purified recombinant NS1 expressed in *Escherichia coli* (400 ng/well) in triplicates. The plates were washed 3 times with phosphate-buffered saline (PBS) containing 0.05% Tween-20 (PBST) and blocked with 1xPBST containing 3% skim milk and 0.1% of BSA for 2 hours at 37°C or 40°C. After a new wash cycle, the anti-NS1 mAb 4F6 [14] was diluted (log2) starting at 157.3 nM, added to wells and incubated at 37°C or 40°C for 2 hours. After a new wash cycle, the anti-mouse IgG antibody conjugated to peroxidase (Sigma, USA) was added to wells and incubated again for 2 hours at 20 ± 2°C. After a final washing cycle, plates were developed with sodium citrate buffer (pH 5.8) containing ortho-phenylenediamine dihydrochloride (Sigma, USA) and H_2_O_2_ and the reaction was stopped after 15 min with the addition of 50 μl of H_2_SO_4_ at 2 M. The optical density reading was performed at 492 nm plate reader (Labsystems Multiscan, Thermo-Scientific, USA).

### ELISA measurements with malarial PvMSP1_19_ antigen and antibody

Recombinant protein PvMSP1_19_ was kept at 37°C, 40°C or 42°C for 1 hour prior to ELISA assays. PvMSP1_19_ was employed as solid phase-bound antigen (200 ng/well) and a volume of 50 μl of each solution was added to each well of 96-well plates (BD Costar 3590). After overnight incubation at each indicated temperature, the plates were washed with PBST and blocked with 5% milk-2.5% BSA for 2 hours, at each specified temperature. The plates were washed with PBST and the monoclonal antibody K_2_3 [12] was tested at serial dilutions (2x) initiating at 93.32 nM in a final volume of 50 μl of sample added to each well in triplicate, followed by incubation for 1 hour at each temperature. After washes with PBST, 50 μl of a solution containing anti-mouse IgG (KPL) conjugated to peroxidase diluted 1:3.000 was added to each well and incubated at 20 ± 2°C for 1 hour. The enzymatic reaction was developed using 3,3´, 5,5´tetramethylbenzidine (TMB) (Bio-Rad) for 15 minutes, and stopped using H_2_SO_4_ 1N. The optical density values were determined at 450 nm. ELISA data was analysed using Prism 7 (GraphPad, USA).

### Circular Dichroism (CD)

CD measurements were performed with a JASCO-810 spectrometer (Jasco, Japan) coupled to a Peltier temperature controller (Model JWJTC-484). Immune complexes were reconstituted at 20 ± 2°C and consisted of 3.37 μM MSP1_19_ in complex with 1.8 μM IgG K_2_3 or 2.7 μM NS1 in complex with 4.27 μM IgG 4H1BC. Three hundred microliters of each complex were immediately thereafter placed in the 1-mm CD cell for 1 hour at specified temperatures, prior to data acquisition. All measurements were performed in 10 mM PBS, pH 7.4 with 1 mM 2-mercaptoethanol. Data were averaged from three scans at 100 nm/min, data pitch 0.1 nm, bandwidth 1 nm. Buffer baselines were subtracted from respective sample spectra.

### Isothermal titration calorimetry (ITC)

#### 1. Sample preparation and setup

Protein concentrations were determined spectrophotometrically by measuring the absorbance at 280 nm with a NanoDrop 2000 (Thermo-Scientific, USA). Molar absorption coefficients for all proteins were calculated with ProtParam (SIB, Switzerland). ITC measurements were performed on a MicroCal iTC 200 calorimeter (GE Healthcare, USA). All direct titrations were performed in the same PBS buffer at 100 mM, pH 7.4, with 1 mM 2-mercaptoethanol that was used for protein dialysis. Control antigen titrations into buffer were subtracted from data using Microcal Origin v7.0 (OriginLab, USA). Data represents averages of 2 or 3 measurements. All measurements comprised of either antigen in syringe at 25 μM (NS1) or 45 μM (MSP1_19_) and 0.9 μM (anti-NS1 IgG1) or 5 μM (anti-MSP1_19_ IgG) in the cell, respectively. Antibodies were kept in the iTC200 cell at each indicated temperature for 1 hour prior to measurements. Antigens were separately heated for the same period in 200 μL Eppendorf microcentrifuge tubes in a water bath (Thermo-Scientific, USA) before start of titrations at same temperatures as the antibodies.

#### 2. Fitting procedures using KinITC (Affinimeter, Spain)

The binding constant (K_A_), the molar enthalpy of the interaction process (ΔH), the molar heat of dilution (ΔH_dil_) and the active titrate concentration correction factor (r_M_), were used as fitting parameters, until the goodness of the fit given by parameter χ^2^ ≤ 1 (for both thermodynamic and kinetic data), thus justifying a 1:1 independent binding sites model. The final value for r_M_ was close to 1 in all thermograms, indicating that the nominal concentration and stoichiometry were correct. The heat capacity changes between 37°C and 42°C were calculated using an integrated form of the Van't Hoff equation for the general case when ΔC_p_ is temperature-dependent. kinITC-ETC (Equilibration time curve) was used to also derive kinetic information from ITC experiments. Fitted response time [seconds] and k_on_ were varied until χ^2^ ≤ 1. K_off_ rates were calculated from Kd = k_off_/k_on_, at each temperature.

#### 3. Statistical analyses

Thermodynamic and kinetic data was first tested for normality, followed by one-way analysis of variance (ANOVA) and a post-hoc analysis with Tukey HSD test, using OriginPro (OriginLab, USA). We compared the data means obtained for the three temperatures of each experimental parameter for either immune complex. We also compared the means of ELISA data obtained at 37°C and 40°C with or without the thermal equilibration step. Calculated powers varied between 0.67 and 0.99 (0.1 for comparison of means for ELISA data at 37°C and 40°C for thermally equilibrated samples). All p-values are shown at 0.05 significance level.

## Results and discussion

### ITC and ELISA measurements

**Fig 1:**
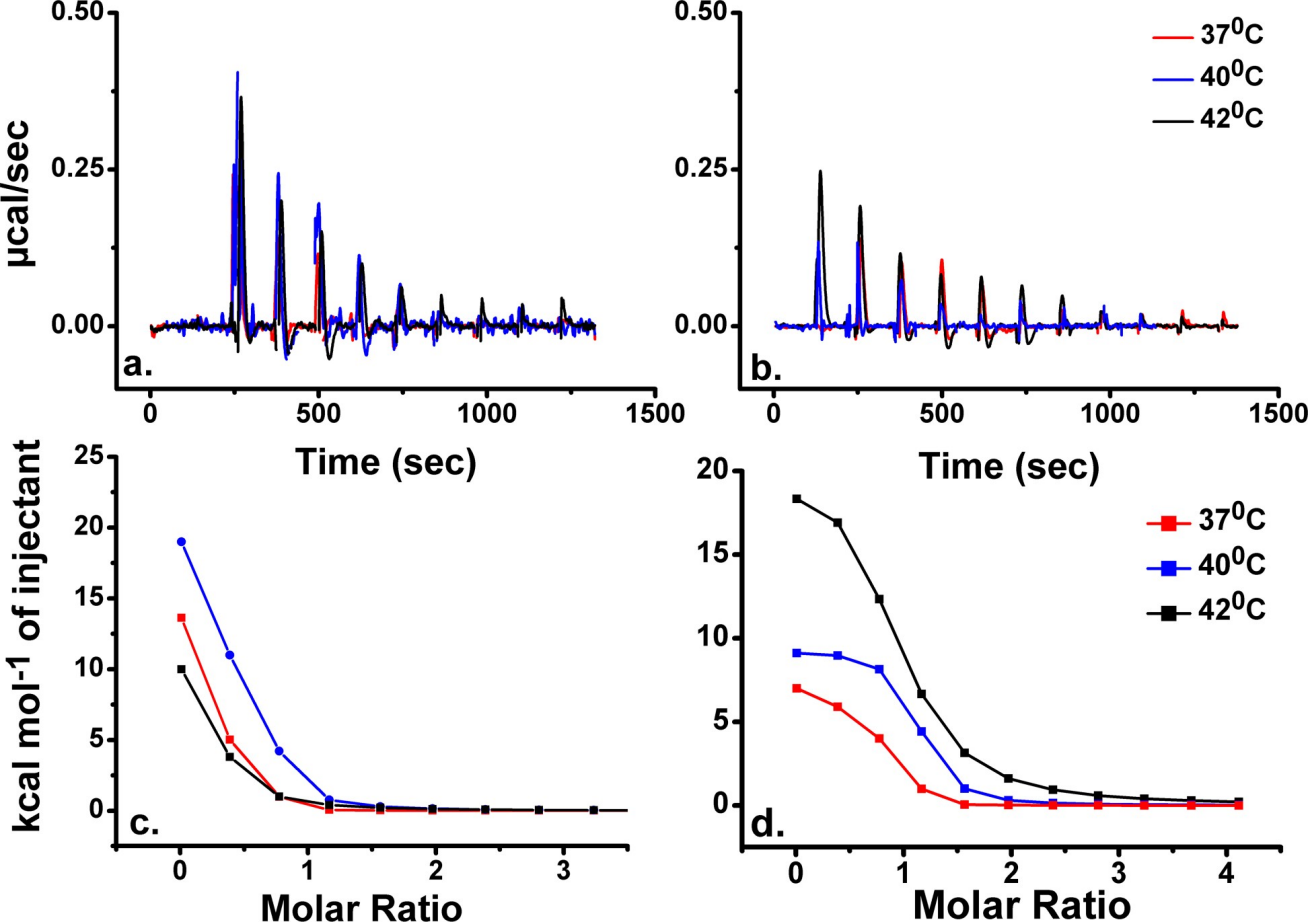
**ITC measurements in PBS buffer of malarial MSP1**_**19**_ **titrated into K**_**2**_**3 IgG antibody (a–raw data, c–binding isotherms) and DENV-2 NS1 titrated into 4H1BC IgG1 antibody (b–raw data, d–binding isotherms) at indicated temperatures.** Assays performed at physiological temperature (37°C, red trace), at fever temperature (40°C, blue trace) and at 42°C (black trace). The heat signals due to binding were obtained as the difference between the heat of reaction and the corresponding heat of dilution.

We used Isothermal Titration Calorimetry (ITC) in order to perform a systematic investigation into the formation of dengue and malarial immune complexes at 37°C, 40°C, and 42°C. We first equilibrated *in vitro* all the proteins for 1 hour at indicated temperatures. Such procedure ensured that the partner proteins were subjected to temperatures more relevant to those caused by fever-inducing pathogens, and for periods that are not lethal *in vivo*, e.g. less than 6 hours at 41°C-42°C for *P*. *vivax* infections [15]. Representative ITC thermograms are shown in Fig 1 (also S2 Fig in Supporting Information).

The changes in enthalpy are different in the two systems, and are characterized by the lowest positive values at 42°C for the malarial complex, following a peak increase at 40°C and then a decrease at 37°C (Fig 1A and 1C); in contrast, for the dengue immune complex, we measured a linear increase of enthalpy with temperature (Fig 1B and 1D).

**Fig 2:**
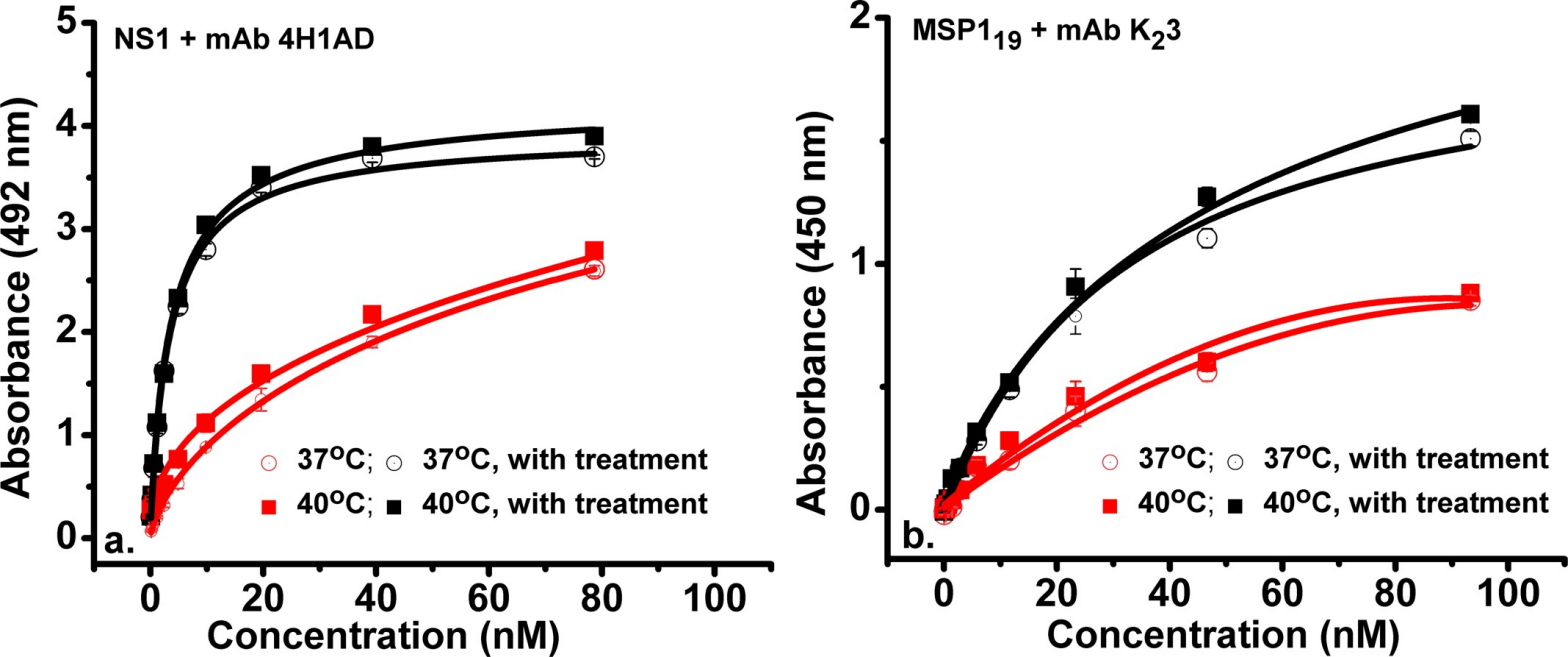
a. ELISA measurements of dengue DENV-2 NS1 antigen with IgG1 4H1BC, with a thermal pre-equilibration step (black square symbol at 40°C, black circle symbol at 37°C) or without this treatment (red square symbol at 40°C, red circle symbol at 37°C). b. ELISA measurements of malarial MSP1_19_ antigen with IgG K_2_3 with a thermal pre-equilibration step (black square symbol at 40°C, black circle symbol at 37°C) or without thermal priming (red square symbol at 40°C, red circle symbol at 37°C). Background-subtracted data represent averages of three independent readings.

We observed that for both systems there was an increase in binding affinity at 40°C that was not observed in other temperatures. This was also measured in solid-phase assay with ELISA, as presented in Fig 2.

For both immune complexes, we observed a ~1.15–1.3 increase in affinity from 37°C to 40°C, in the absence of thermal priming, according to ELISA measurements. Thermal pre-equilibration led to significant improvements in K_D_ by a factor of ~9.5 (at 37°C) and ~8 (at 40°C) for the dengue immune complex, and to a decrease in K_D_ by a 1.3 factor (at 37°C) and a 1.2 factor at 40°C for the malarial complex, respectively. An overview of ITC and ELISA results, together with statistical analyses is shown in Table 1.

**Table 1:** Thermodynamic and kinetic data of dengue immune complexes.

Parameters	Dengue complex (37°C)	Dengue complex(40°C)	Dengue complex(42°C)	p-value
K_D_ [nM] (ELISA)	33.3 ± 2.8	25.4 ± 4.1	12.3 ± 1.3	3.5^−5^
K_D_ [nM] (ELISA)*	3.5 ± 0.2	3.1 ± 0.2	NA	0.4
K_D_ [nM] (ITC)**	19.5 ± 0.5	4.9 ± 0.4	36.1 ± 2.4	1.8^−6^
ΔH [kcal/mol]	7.5 ± 0.8	9.1 ± 0.8	18.8 ± 0.9	4.1^−4^
T·ΔS [kcal/mol]	17.1 ± 1.9	35.8 ± 1.4	29.5 ± 1.8	2^−3^
ΔG [kcal/mol]	-9.6 ± 0.5	-26.7 ± 0.3	-10.7 ± 0.4	3.9^−4^
k_**on**_ [10^6^ M^-1^∙s^-1^]	2.1 ± 0.3	0.43 ± 0.13	2.73 ± 0.24	5.9^−5^
k_**off**_[s^-1^]***	0.04	0.0022	0.1	4^−2^
ΔC_**p**_ [kcal/mol·K]	3.7 ± 0.3		

*Values for thermally pre-equilibrated samples. NA–data not available.

**Standard deviations (s.d.) between 15–25%. Values are averages with s.d. of three measurements (two measurements for thermograms at 42°C). P-values represent comparisons between the data means obtained for the three temperatures of each experimental parameter.

ELISA did not yield statistically significant differences in data between the thermally pre-equilibrated dengue complexes at 37°C and 40°C (Table 1). Statistical differences were calculated between the thermally pre-equilibrated and non-thermally primed ELISA samples (p-value of 5.9^−6^ for measurements at 37°C, and a p-value of 1.3^−5^ at 40°C, respectively). Previous *in vitro* non-calorimetric measurements of antibody-antigen complex formation at increasing temperatures reported either a very limited variation in the values of the equilibrium constants from 25°C to 40°C [16, 17], or even a decrease thereof from 2°C to 40°C [18, 19].

In contrast, our ITC measurements for the dengue system at 40°C revealed a K_D_ that was lower by a factor of ~4 compared to values at 37°C and by a factor of ~7, if compared to measurements at 42°C, respectively. The main contribution to binding affinity is entropic, without compensation by the large positive enthalpy variations, as previously described for other antibody-antigen complexes [20]. The intrinsic free energy of binding peaking at 40°C was largely independent of temperature variations or of the entropic/enthalpic terms [21]. Kinetic parameters that depend directly on temperature were largely responsible for the differences in affinities observed across the temperature range, especially with the most favorable k_off_ rates measured at 40°C.

Epitope-mapping studies have identified the sequence ^193^AVHADMGYWIESALNDT^209^ as the conformational epitope from a β-sheet structure of the dengue NS1 protein [22]. The presence therein of three negative charges conferred by the aspartic and glutamic acid residues may result in more stable immune complexes at higher temperature, as previously measured in other systems [23]. It is significant to note that the enthalpic contribution to binding is not negligible, providing a basal binding affinity to which the entropic factor is added, brought on by increased orientational disorder of water molecules at higher temperatures. Importantly, the effect of temperature changes in modulating monoclonal antibodies binding to other DENV-2 antigens was also shown to be important for its structural E proteins, where unique quaternary conformational epitopes were exposed when virions were incubated at vertebrate host physiological temperature (37°C), compared to the mosquito thermal optimum of 28°C-30°C [24].

ITC measurements for the malarial complex yielded a K_D_ approximately ~6 and ~10 fold lower at 40°C when compared to values at 37°C and at 42°C, respectively (Table 2).

**Table 2:** Thermodynamic and kinetic data of malarial immune complexes.

Parameters	Malarial complex (37°C)	Malarial complex(40°C)	Malarial complex(42°C)	p-value
K_D_ [nM] (ELISA)	51.2 ± 2.4	39.9 ± 3.3	47.8 ± 2.8	4.1^−2^
K_D_ [nM] (ELISA) *	41.4 ± 3.2	35.3 ± 1.7	NA	1.1^−3^
K_D_ [nM] (ITC) **	22.2 ± 1.6	3.8 ± 0.3	43.6 ± 1.9	1.4^−4^
ΔH [kcal/mol]	16.5 ± 1.3	20.1 ± 2.3	8.9 ± 1.7	7.4^−4^
T·ΔS [kcal/mol]	24.7 ± 1.6	32.6 ± 1.4	12.6 ± 2.4	1.6^−3^
ΔG [kcal/mol]	-8.2 ± 0.2	-12.5 ± 0.9	-3.6 ± 0.4	6.1^−3^
k_**on**_ [10^6^ M^-1^∙s^-1^]	1.9 ± 0.4	0.65 ± 0.15	4.2 ± 0.6	4.3^−3^
k_**off**_[s^-1^]***	0.036	0.002	0.17	9.8^−3^
ΔC_**p**_ [kcal/mol·K]	0.97 ± 0.23		

*Values for thermally pre-equilibrated samples. NA–data not available.

**Standard deviations (s.d.) between 15–25%. Values are averages with s.d. of three measurements (two measurements for thermograms at 42°C). P-values represent comparisons between the data means obtained for the three temperatures of each experimental parameter.

Unlike the results obtained with the dengue system, we calculated statistical differences between the thermally pre-equilibrated and non-thermally primed ELISA samples, with a p-value of 4.5^−4^ for measurements at 37°C, and a p-value of 1.7^−2^ at 40°C, respectively (Table 2).

The peak in affinity at 40°C is, similar to the DENV-2 system, due to favorable k_off_ rates if compared to those observed at 37°C and at 42°C. This is similar to what occurs with DENV2 system. At higher temperatures, the entropic factor becomes the primary contributor to binding, suggesting the occurrence of considerable solvation effects and/or enhanced antibody-antigen flexibility upon complex formation. The reduced role of enthalpy of hydration in binding reaction, in contrast to the entropic contribution upon temperature increase has previously been observed for hapten-antibody and other antigen-antibody complexes [16, 25]. For both systems and especially for the dengue immune complex, we have measured a large positive heat capacity change that also adversely affects binding affinity, which is indicative of the onset of unfolding, and is analogous to temperature-activated TRP channels [26]. The heat capacity change is characterized not only by the hydrophobic effect, which is due to changes in hydration of nonpolar binding surfaces upon temperature increase [27, 28], but also by changes in electrostatic interactions and hydrogen bonding [28], resulting in the positive ΔH we measured.

We propose that at physiological temperatures the formation of these immune complexes is energetically less favored, if an encounter step and a subsequent docking step for these protein partners are envisaged. While temperature increase favors partner encounter, at higher non-denaturing temperatures (e.g.: 40°C), a large proportion of the immune complexes may anneal to a more stable docked state [29]. This latter step may be optimized during affinity maturation of the antibodies, given the intracellular thermal gradient [30], and may result in differential activation of various temperature-sensitive proteins [31].

Using Circular Dichroism, we did not detect significant secondary structure modifications in any protein partner or complex, across the temperature range here used (S1 Fig). The differences in affinities that we detected between the two techniques have previously been reported for a malarial system involving MSP1 [32]. These discrepancies may be accounted for by the presence of the adsorbed phase in ELISA measurements that could hinder epitope binding, producing steric or attractive interactions between the mAb molecules [33] or inducing blocking of binding sites by multivalent binding and rebinding [34], which ultimately affect association rates. These restrictions may be reduced for thermally equilibrated ELISA samples, resulting in higher affinities if compared to ITC results or non-equilibrated ELISA data (Tables 1 and 2).

In conclusion, our data indicate the potential for reversible, physiological fever temperatures in increasing *in vitro* antibody affinity for tertiary and quaternary epitopes and suggest a thermal activation step for fever antibodies binding to antigens. This new role may constitute an important adaptive mechanism for antibody-mediated detection and protection against pathogens. Further validation by *in vivo* studies and extension to a larger set of antigens involved in fever episodes, including from bacterial pathogens, will extend the reach of our conclusions. In addition, our results may add to the growing interest in relating hyperthermia to the efficiency of cancer immunotherapy [35]. Finally, thermal equilibration of the protein partners prior to performing ELISA or other relevant *in vitro* assays may improve the binding affinities and inform on the appropriate temperature conditions of the testing environment, thus assisting in cases where limited amounts of samples are available.

## Supporting information

S1 Fig Circular Dichroism data at each indicated temperature. Background-subtracted CD data of a. malaria MSP1_19_ antigen in immunocomplex with IgGK_2_3and b. DENV-2 NS1 antigen in complex with IgG 4H1BC, at specified temperatures.
(TIF)

S2 Fig Supplementary ITC thermograms. ITC measurements of malarial and dengue complex formation at indicated temperatures. ITC measurements of DENV-2 NS1 titrated into 4H1BC IgG1 antibody (a,c) and malaria MSP1_19_ titrated into K_2_3 IgG antibody (b,d) at indicated temperatures.
(TIF)
