# Supplementary figures and images for: Febrile temperatures increase in vitro antibody affinity for malarial and dengue antigens
Source: PLoS Negl Trop Dis. 2019 Apr 3;13(4):e0007239. doi: 10.1371/journal.pntd.0007239 (PMC6464238; doi:10.1371/journal.pntd.0007239)

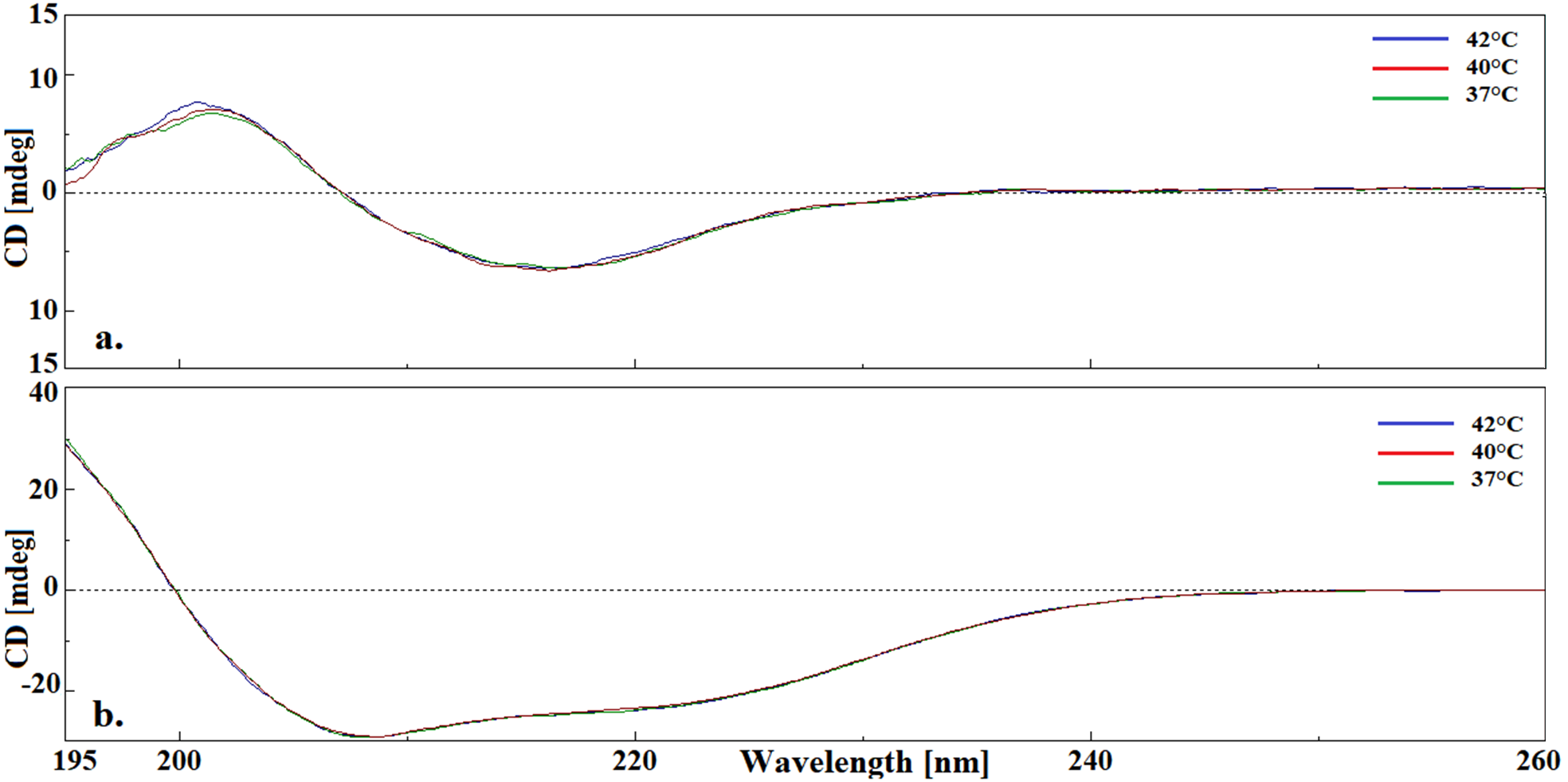

Supplement: S1 Fig — Background-subtracted CD data of a. malaria MSP119 antigen in immunocomplex with IgGK23and b. DENV-2 NS1 antigen in complex with IgG 4H1BC, at specified temperatures. (TIF) [file pntd.0007239.s001.tif]

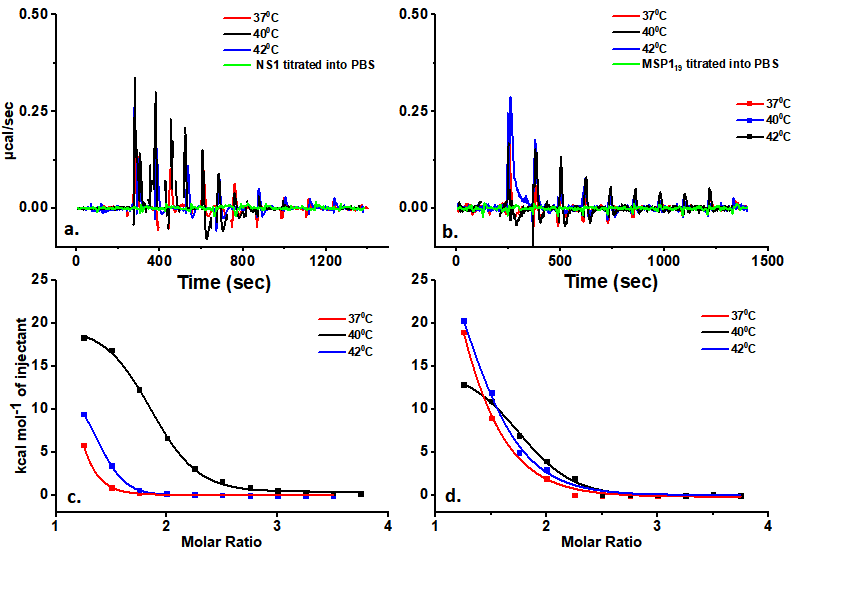

Supplement: S2 Fig — ITC measurements of malarial and dengue complex formation at indicated temperatures. ITC measurements of DENV-2 NS1 titrated into 4H1BC IgG1 antibody (a,c) and malaria MSP119 titrated into K23 IgG antibody (b,d) at indicated temperatures. (TIF) [file pntd.0007239.s002.tif]
